# Life cycle and population growth rate of *Caenorhabditis elegans *studied by a new method

**DOI:** 10.1186/1472-6785-9-14

**Published:** 2009-05-16

**Authors:** Daniel Muschiol, Fabian Schroeder, Walter Traunspurger

**Affiliations:** 1 Animal Ecology, University Bielefeld, Morgenbreede 45, 33615 Bielefeld, Germany

## Abstract

**Background:**

The free-living nematode *Caenorhabditis elegans *is the predominant model organism in biological research, being used by a huge number of laboratories worldwide. Many researchers have evaluated life-history traits of *C. elegans *in investigations covering quite different aspects such as ecotoxicology, inbreeding depression and heterosis, dietary restriction/supplement, mutations, and ageing. Such traits include juvenile growth rates, age at sexual maturity, adult body size, age-specific fecundity/mortality, total reproduction, mean and maximum lifespan, and intrinsic population growth rates. However, we found that in life-cycle experiments care is needed regarding protocol design. Here, we test a recently developed method that overcomes some problems associated with traditional cultivation techniques. In this fast and yet precise approach, single individuals are maintained within hanging drops of semi-fluid culture medium, allowing the simultaneous investigation of various life-history traits at any desired degree of accuracy. Here, the life cycles of wild-type *C. elegans *strains N2 (Bristol, UK) and MY6 (Münster, Germany) were compared at 20°C with 5 × 10^9 ^*Escherichia coli *ml^-1 ^as food source.

**Results:**

High-resolution life tables and fecundity schedules of the two strains are presented. Though isolated 700 km and 60 years apart from each other, the two strains barely differed in life-cycle parameters. For strain N2 (*n *= 69), the intrinsic rate of natural increase (*r*_m_d^-1^), calculated according to the Lotka equation, was 1.375, the net reproductive rate (*R*_0_) 291, the mean generation time (*T*) 90 h, and the minimum generation time (*T*_min_) 73.0 h. The corresponding values for strain MY6 (*n *= 72) were *r*_m _= 1.460, *R*_0 _= 289, *T *= 84 h, and *T*_min _= 67.3 h. Peak egg-laying rates in both strains exceeded 140 eggs d^-1^. Juvenile and early adulthood mortality was negligible. Strain N2 lived, on average, for 16.7 d, while strain MY6 died 2 days earlier; however, differences in survivorship curves were statistically non-significant.

**Conclusion:**

We found no evidence that adaptation to the laboratory altered the life history traits of *C. elegans *strain N2. Our results, discussed in the light of earlier studies on *C. elegans*, demonstrate certain advantages of the hanging drop method in investigations of nematode life cycles. Assuming that its reproducibility is validated in further studies, the method will reduce the inter-laboratory variability of life-history estimates and may ultimately prove to be more convenient than the current standard methods used by *C. elegans *researchers.

## Background

Since 1965, when Sydney Brenner chose the free-living nematode *Caenorhabditis elegans *(Maupas, 1900) Dougherty, 1953 as the model animal in which to investigate development and function in a simple nervous system, an enormous amount of work has been done on "the worm". Despite the practical challenge of being tiny to work with at about 1 mm in length, *C. elegans *is easy to breed and maintain, in addition to having a short generation time and lifespan. These features have made *C. elegans *the predominant model organism in biological research. Today, the depth of understanding of the genetics, anatomy, and developmental biology of this organism probably exceeds that of any other animal [1] and it remains the only metazoan in which the entire cell lineage (959 cells) has been traced, from egg to adult [2].

A wide variety of studies have reported life-history traits (LHTs) of *C. elegans*. Such traits include juvenile growth rates, age at sexual maturity, adult body size, age-specific fecundities, total reproduction, generation time, age-specific mortality, mean and maximum lifespan, and the intrinsic rate of natural increase (*r*_m_: see [3]). Investigations evaluating the LHTs of *C. elegans *have covered many different aspects, such as inbreeding depression and heterosis [4-7], dietary restriction/supplement [8-12], mutations [13-17], ecotoxicology [18-21], and ageing (review in: [22]).

In reviewing the relevant literature, we found that the many excellent studies on *C. elegans *make use of quite different approaches in the design of life-cycle experiments. But the high diversity of protocol designs (cultivation temperature, solid vs. liquid media, food quality and quantity, way of data acquisition) hampers comparisons of data from different sources. For example, probably as a result of differing protocol designs, values of *C. elegans *wild-type total fecundity in the literature differ by a factor of up to 8.6 (327 vs. 38: see [10,23]). Moreover, data collected under obviously suboptimal culture conditions may be difficult to reproduce.

In an attempt to join the advantages of liquid and solid media in the cultivation of nematodes, we recently developed an easy, fast, and yet precise method to perform life-cycle experiments [24]. In the present work, we adopted this 'hanging-drop' method to studies of the wild-type *C. elegans *strain N2. The method's convenience and reliability make its application of interest in many *C. elegans*-related research fields.

Additionally, we aimed to iterate a recent study by Chen et al. [25] which provided evidence that strain N2 has adapted to laboratory conditions with respect to many important demographic parameters. This *C. elegans *strain had been in continuous laboratory culture since the 1940s [6] before it became the geneticists' reference wild-type strain N2 [26]. The influence of inadvertent selection and genetic drift on *C elegans *strains kept in culture is unclear [25]. Since, we compared the N2 strain to a recently isolated strain, MY6, from Münster (Northwest Germany) in order to determine whether the two strains, isolated 700 km and 60 years apart from each other, differed with respect to several LHTs. The specific aim of the study was to supplement existing data on *C. elegans *with complete high-resolution life tables and fecundity schedules and to assess the nematode's age at sexual maturity, mean and maximum lifespan, net reproductive rate (*R*_0_), total fertility rate (*TFR*), generation time, population doubling time, and intrinsic rate of increase (*r*_m_).

## Methods

Two wild-type isolates of *Caenorhabditis elegans*, MY6 and N2, were obtained from the Caenorhabditis Genetics Center (University of Minnesota, St Paul) on NGM (Nematode Growth Medium: see [27]) agar plates spotted with OP50 (a uracil-requiring mutant of *E. coli*). Strain MY6 had been isolated in July 2002 from a compost heap in Roxel, Münster (Northwest Germany) and frozen within five generations after isolation by H. Schulenberg (Westphalia Wilhelm's University, Münster). Strain N2, isolated from mushroom compost near Bristol (UK) by L.N. Staniland, is the canonical 'wild-type' *C. elegans *strain used in laboratories throughout the world. It has been maintained in the laboratory (interrupted by periods of freezing) for about 60 years.

### Experimental set-up

Three weeks prior to the life-cycle experiments, the two *C. elegans *strains were transferred to NGG (Nematode Growth Gelrite: see [24]) culture plates seeded with OP50 in order to remove the influence of maternal effects. The preparation and ingredients of NGG are analogous to those of standard NGM, the only modification being the replacement of Bacto-agar by 1.5 g l^-1 ^gellan gum, a bacterial exopolysaccharide (Gelrite, Merck & Co., Kelco Division) [28]. The two strains were kept in the exponential growth phase by sub-culturing them onto fresh culture plates every 6 days. Stocks were kept at 20.0°C and life-cycle experiments were carried out at the same temperature. All manipulations were done at room temperature (20 ± 1°C).

The original description of the experimental procedures used to record the reproductive output and lifespan of individual worms can be found in Muschiol & Traunspurger [24]. Briefly, synchronous stage 1 juveniles (J1) of *C. elegans *strains N2 and MY6 (*n *= 72 per strain) were kept individually in hanging 8-μl drops of food medium in the lid of 12-well multiwell plates (Greiner 665102). The food medium consisted of washed *E. coli *OP50 cells resuspended in semi-fluid NGG. The gel-like consistency of the medium was achieved by constant stirring of freshly autoclaved NGG on a magnetic stirrer during the cooling period. This food medium is perfectly suited for life-cycle experiments as its viscosity permits the nematodes to move freely but prevents bacterial cells from accumulating at the bottom of the drop.

Bacterial density was set to 5 × 10^9 ^cells ml^-1 ^with reference to a previously determined absorption (OD_600_) vs. cell density curve [24]. The chosen cell density was well-considered, as it is critical in life-cycle experiments to preclude food limitation. Since Schiemer [29], working with *Caenorhabditis briggsae*, reported a somewhat reduced fecundity at food concentrations of 10^9 ^*E. coli *cells ml^-1^, we chose to provide a higher concentration of bacteria. However, very high food concentrations may have a detrimental effect, as shown by Johnson et al. [30], who reported a dramatically reduced mean life expectancy of *C. elegans *at *E. coli *densities of 10^10 ^cells ml^-1^.

As soon as the ovaries of the experimental animals began to develop (J4), each individual was transferred to a fresh drop of food medium every 6–24 h (see below) while the previous drop was checked for produced offspring. In *C. elegans*, it is not sufficient to determine offspring in terms of produced eggs because self-progeny brood sizes are determined by the number of self-sperm and the additional oocytes produced are laid unfertilized unless the hermaphrodite is mated [23]. In order to distinguish between sterile and fertile eggs, each transfer of a maternal worm to a fresh food drop was followed by a 24-h-incubation of the previous drop. After this period, all fertile eggs had hatched. The juveniles were relaxed by heating the drop to 80°C and then fixed and stained by the addition of an 8-μl drop of 37% formaldehyde and Rose Bengal (300 μg ml^-1^). Then, the fixed samples were covered with a circular 18-mm cover slip and counted under a dissecting microscope at 40-fold magnification, using an underlying grid to facilitate counting. The experiment was conducted until the last adult died (day 32). A worm was scored as dead if it ceased to respond to light touch with an eyelash mounted on the tip of an applicator stick and showed a loss of turgor. Dead worms were kept for an additional 24 h, after which they were checked for offspring hatched within the mothers' carcasses. Individuals that were lost during handling were excluded from the data set (*n *= 3 or 2.1% of total individuals).

### Time Resolution

A species' intrinsic rate of population increase is determined to a much greater extent by the rate of oviposition in the first days of adult life than by the total number of eggs laid in the lifespan of the adult. With each successive day, the contribution of eggs laid to the value of *r*_m _is less [3]. Accordingly, in the experimental determination of oviposition rates, those measured in early adult life should be the most accurate. When reproduction is continuous, as it is in *C. elegans *populations, the continua of time *t *and age *x *can be divided into discrete intervals of arbitrary length, thus approximating a continuous-time model to any desired degree of accuracy by a discrete age-class model ([31]: p. 14). In nematodes, the length of each age class is usually set to 1 day (e.g., [29]). Since *C. elegans *is an extremely fast-developing species that matures in less than 72 h, age classes of 1 day appeared quite broad to us. Thus, instead, we increased the accuracy by determining oviposition rates every 6 h during early adult life. In order to keep workload on a reasonable scale (576 drops of food medium had to be fixed and checked for offspring daily), temporal resolution was reduced to 12 h after age-class *x *= 128.5 (h) and to 24 h after *x *= 293.5. Additionally, the number of replicates was reduced from 72 to 36 per strain after *x *= 137.5, i.e., when more than 90% of total reproduction was completed.

### Creation of synchronous cohorts

To obtain precise fecundity schedules, it is crucial to perform life-cycle experiments with a cohort of highly synchronous individuals. Then, age-specific fecundities are determined between two identical stages of successive generations (egg-egg/J1-J1). In *C. elegans*, however, the time of egg deposition is a poor indicator of the egg's developmental stage, as the time from egg fertilization to egg-laying can vary considerably depending on the physiological condition and ontogenetic stage of the animal to be assayed ([32]: p. 157). Egg-laying can even be delayed to the extent that hatching occurs within the uterus ("bagging" or matricidal hatching: see [33]) or instantly after the eggs have been laid. Accordingly, the time of hatching rather than the time of egg deposition is a better indicator of ontogenetic age [4]. Thus, we started our experiment with cohorts of juveniles that had hatched within a narrow time span (less than 4 h). However, the experimental set-up applied in this investigation recorded fecundity in terms of fertile eggs per time interval (see above). In order to account for this discrepancy (start of the experiment with juveniles, but offspring counted as eggs), we determined the average time required by a freshly laid egg to hatch (*T*_hatch_). The (virtual) starting point of our experiment (age *x *= 0) was then defined as the hatching time of the examined juveniles minus *T*_hatch_.

### Estimation of hatching time *T*_hatch_

Adults and stage 4 juveniles of both *C. elegans *strains were randomly picked from exponentially growing NGG culture plates and transferred to 20-μl drops of semi-fluid NGG containing OP50 at a density of 5 × 10^9 ^cells ml^-1^. Ten individuals were pooled in one drop; ten drops per strain were prepared and incubated at 20.0°C. After 15 h, the drops were fixed, stained, and examined under a dissecting microscope at 40-fold magnification. An underlying grid facilitated counting of the eggs (*N*_egg_) and hatched juveniles (*N*_juv_). The average hatching time was calculated as the proportion of unhatched eggs to total eggs laid multiplied by the experimental time (*T *= 15 h): *N*_egg_/(*N*_egg_+*N*_juv_) × *T*. If, for example, one third of all laid eggs had been hatched, it was assumed that these hatched eggs had been laid, on average, during the first third of the 15 h whereas all eggs laid in the last two thirds did not have enough time to hatch – resulting in an average hatching time of 10 h. *T*_hatch _was calculated separately for each strain as the arithmetic mean of the 10 drops per strain.

### Data processing

Life tables and fecundity schedules are difficult to interpret on their own because they hide the dynamic behaviour of a population behind a mass of detail ([34]: p. 151). For this reason, the data were summarized according to the upper-order parameters generation length, doubling time, and intrinsic rate of natural increase (*r*_m_) using the fundamental equation of population dynamics:



This equation is called the Euler equation, ([31]: p. 23) and is also frequently referred to as the Lotka equation, after Lotka [35], who applied it to human demography. As the equation does not lend itself to a direct solution, it has to be estimated by iteration (substituting successive trial values of *r*_m _in the equation until the left-hand side sums to 1).

The intrinsic rate of natural increase (*r*_m_) is the growth rate of a population that has a stable age distribution and grows in an unlimited environment. Since *r*_m _integrates the entire age schedules of survival and fertility into a single measure, it measures fitness in age-structured populations [31]. In this study, the high time resolution of 6–24 h per age-class and the consequential extensive life tables made it necessary to determine *r*_m _using a Microsoft^® ^Visual Basic (6.0) macro in Excel (2007), which may be obtained from the authors on request. The net reproductive rate (*R*_0 _= ∑*l*_x_*m*_x_) is defined as the average number of offspring that an individual in a population will produce in its lifetime. Unlike the total fertility rate (*TFR*), *R*_0 _depends on age-specific mortality rates. Since the concept of generation time is considered as rather arbitrary and slippery in the context of age-structured populations, several alternative measures have been proposed (for a general survey, see [31]). Here, the values *T*_0_, *T*_1_, and *T *were compared, as defined by the following equations:



*T*_0 _(also referred to as *T*_c_, the cohort generation time) is the mean age at reproduction of a cohort of females. *T*_1 _refers to that period of time necessary for a population growing at a constant rate *r*_m _to increase by the factor *R*_0_. *T *is the mean age of the mothers of a set of newborn individuals in a population with a stable age distribution.

To analyse discrete and non-paired data, a non-parametric Mann-Whitney U-test or, when appropriate, parametric T-test was used. The assumption of homogeneous variances in the T-test was confirmed with Brown & Forsythe's test. The survival of the two *C. elegans *strains was compared using the log-rank test. Total numbers of fertile eggs were correlated with total lifespan using Spearman's rank correlation coefficient. All statistical analyses were carried out using the Statistica software package (v7.0, StatSoft Inc. 2004).

## Results

### Hatching

The comparison of hatching times (time from egg deposition to hatch) revealed a small but significant (*d.f*. = 38, *t *= -5.2, *p *< 0.001) difference between the two *C. elegans *strains: At 20.0°C, freshly laid eggs of strain N2 needed, on average, 7.3 h to hatch (range 5.0 – 10.6; Table 1) while eggs of strain MY6 needed 9.9 h (range 6.8 – 11.9).

**Table 1:** Life-cycle parameters of *C. elegans *strains MY6 and N2

	N2	MY6	*p*-level
			
*T*_hatch _[h]	7.3 ± 1.6	9.9 ± 1.5	< 0.001
lifespan [d]	16.7 ± 5.8	14.7 ± 4.9	0.11
*T*_min _[h]	73.0 ± 4.4	67.3 ± 3.1	< 0.001
*T*_rate _[h]	108.2 ± 9.0	105.5 ± 9.2	0.08
rate [N d^-1^]	141 ± 19	144 ± 22	0.42
*T*_0_/*T*_1_/*T *[h]	115/99/90	106/93/84	-
*r*_m _d^-1^	1.375	1.460	-
PDT [h]	12.1	11.4	-

Values represent arithmetic means ± s.d. All time intervals were measured from the moment of egg deposition.

***T*_hatch _**= time from egg deposition to hatching; ***T*_min _**= age at first egg deposition; ***T*_rate _**= age at maximum rate of egg-laying; **rate **= maximum rate of egg-laying; **total **= total number of eggs produced by individual worms; ***T*_0_**, ***T*_1_**, ***T ***= alternative measures of generation time; ***r*_m _**= intrinsic rate of natural increase; **PDT **= population doubling time

### Survivorship

**Figure 1:**
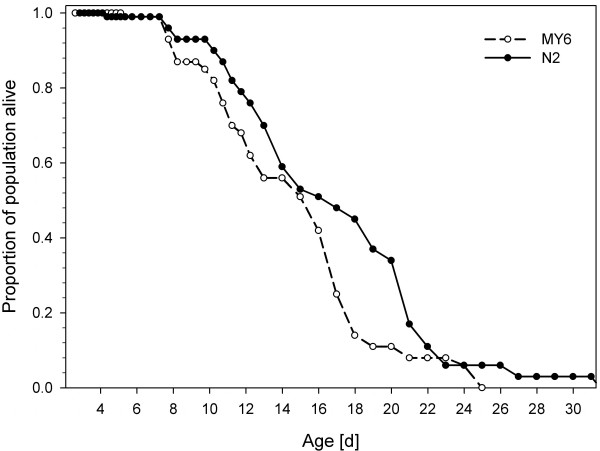
**Hermaphrodite survivorship curves of *C. elegans *strains MY6 and N2**. Lines represent fraction surviving at the given interval after egg deposition (age x = 0). Note that mortality among juveniles and young adults was virtually zero. Population sizes were *n *= 72 (MY6) and *n *= 69 (N2) until age 5.7 d and *n *= 36 (MY6 and N2) thereafter.

Juvenile and early adulthood mortality was negligible in both strains: All 141 juveniles observed in this investigation reached sexual maturity and 99% of them reached an age older than 1 week (173.5 h, Table 2). At that point of time, reproduction was mostly completed, as 93.4% (N2) and 97.5% (MY6) of total reproductive output had already been laid. Plotting survivorship against time (Figure 1) generated type I survivorship curves [36] typical of species with low mortality rates until near the end of the lifespan. During most of the experiment, strain N2 evinced a somewhat lower mortality than strain MY6, as reflected by a slower flattening of its survivorship curve. However, the log rank test revealed no significant difference between them (*z *= 1.68; *p *= 0.09).

**Table 2:** Abbreviated life table and fecundity schedule of the reproductive periods of *C. elegans *strains MY6 and N2

		N2			MY6		
			
x	D	l_x_	m_x_	*l*_x_*m_x_	l_x_	m_x_	*l*_x_*m_x_
62.5	6				1.00	1.04	1.04
68.5	6	1.00	2.93	2.93	1.00	11.56	11.56
74.5	6	1.00	11.30	11.30	1.00	18.36	18.36
80.5	6	1.00	17.77	17.77	1.00	21.36	21.36
86.5	6	1.00	23.45	23.45	1.00	24.99	24.99
92.5	6	1.00	26.38	26.38	1.00	30.24	30.24
98.5	6	1.00	28.49	28.49	1.00	31.10	31.10
104.5	6	0.99	32.04	31.72	1.00	26.47	26.47
110.5	6	0.99	29.81	29.51	1.00	30.69	30.69
116.5	6	0.99	26.78	26.51	1.00	26.10	26.10
122.5	6	0.99	25.07	24.82	1.00	18.85	18.85
128.5	6	0.99	20.09	19.89	0.99	14.34	14.20
137.5	12	0.99	19.35	19.16	0.99	12.92	12.79
149.5	12	0.99	6.17	6.11	0.99	7.17	7.10
161.5	12	0.99	3.34	3.31	0.99	5.03	4.98
173.5	12	0.99	2.94	2.91	0.99	2.60	2.57
185.5	12	0.96	2.85	2.74	0.93	3.03	2.82
197.5	12	0.93	2.12	1.97	0.87	1.48	1.29
209.5	12	0.93	1.27	1.18	0.87	0.81	0.70
221.5	12	0.93	2.88	2.68	0.87	0.81	0.70
233.5	12	0.93	2.58	2.40	0.85	0.37	0.31
245.5	12	0.90	1.72	1.55	0.82	0.15	0.12
257.5	12	0.87	1.61	1.40	0.76	0.37	0.28
269.5	12	0.82	1.28	1.05	0.70	0.24	0.17
504.6			3.09	1.84		0.10	0.06
			
Σ			295	291		290	289

Pivotal time intervals x are related to the average time of egg deposition. Population sizes were n = 69 (N2) and n = 72 (MY6) until x = 137.5 h and n = 36 (MY6 and N2) thereafter.

***x ***= pivotal age in hours; ***D ***= length of age class in hours; ***l*_x _**= age-specific survival probability; ***m*_x _**= age-specific fecundity. ∑***m*_x _**= total fertility rate (*TFR*); ∑*l*_x_*m_x _= net reproductive rate (*R*_0_).

Although maximum lifespan is a poor statistical variable, it is included here because of its wide use as a species-specific parameter in the literature on ageing [37]. The longest-lived individual of strain N2 died on day 32, while individuals of strain MY6 reached a maximum age of 25 days. In contrast to maximum lifespan, mean lifespan is a reliable quantitative marker for the study of mutations and genetic lines that extend life in *C. elegans *([5], and references therein). In the present study, strain N2 lived for 16.7 days, while strain MY6 died 2 days earlier (14.7; Table 1); this difference was statistically non-significant (*d.f*. = 70, *t *= -1.62, *p *= 0.11).

### Reproduction

**Figure 2:**
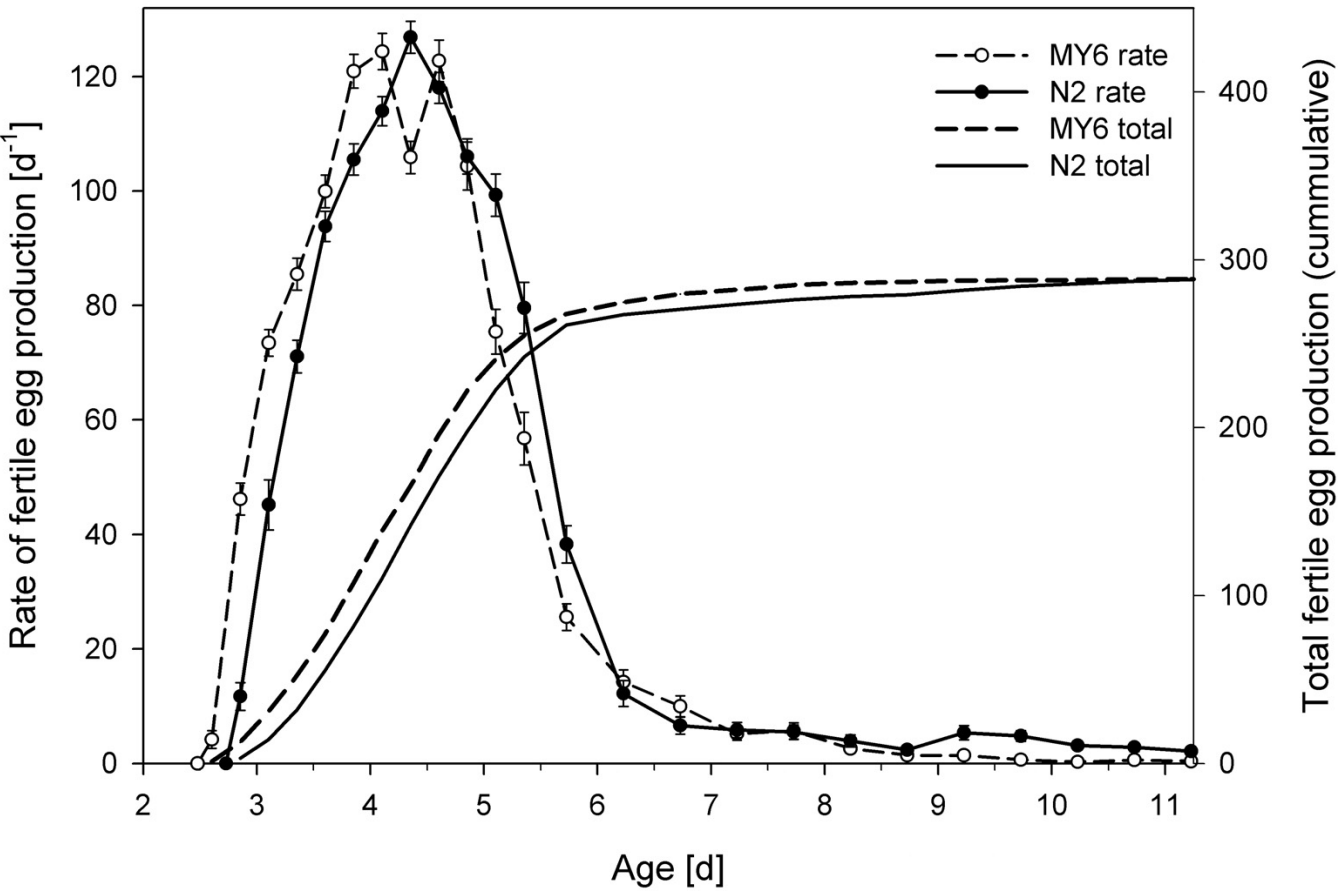
**Fecundities of *C. elegans *strains MY6 and N2**. Population sizes were *n *= 72 (MY6) and *n *= 69 (N2) until age 5.7 d and *n *= 36 (MY6 and N2) thereafter (s.e. bars).

A total of 2832 data points were obtained from the experiment, and a total of 39,075 offspring were counted to obtain the productivity data. Figure 2 shows the fecundities of both *C. elegans *strains at 20.0°C. Strain N2 laid the first eggs at a mean age of 73.0 h (range 68.5 – 80.5; *n *= 69), whereas with a minimum generation time of only 67.3 h (range 62.5 – 74.5; *n *= 72), strain MY6 matured 6 h faster (Table 1). The difference between these minimum generation times (*T*_min_: see [38]) was statistically significant (*U *= 897; *p *< 0.001).

Once reproduction had begun, the egg-laying rate steeply increased, reaching a maximum at an average age of 108.2 h (N2) and 105.5 h (MY6) (Table 1). The maximum egg-laying rates observed in this investigation were 141 (N2) and 144 (MY6) eggs d^-1^. Neither maximum egg-laying rate (*d.f*. = 139, *t *= 0.80, *p *= 0.42) nor the age of the nematode when the former was reached (*d.f*. = 139, *t *= -1.75, *p *= 0.08) differed significantly between the two strains. Interestingly, the egg-laying rate of strain MY6 showed a bipartite maximum, interrupted by a slightly decreased egg-laying rate (Figure 2). The total number of fertile eggs produced during the entire lifespan was 291 for strain N2 and 289 for strain MY6. As suggested by Davies & Hart [10], the total number of produced eggs was plotted against total lifetime in order to estimate whether the two measures were related to each other. In both strains, the distribution appeared rather random (not shown here). In strain N2, Spearman's rank correlation coefficient was not significantly different from zero (*r*_s _= 0.15, *p *= 0.38), indicating neither positive nor negative correlation. In strain MY6, a weak but significant positive correlation was found (*r*_s _= 0.44, *p *< 0.01).

The values of the three alternative measures of mean generation time, *T*_0_, *T*_1_, and *T*, are given in Table 1. According to Charlesworth [31], *T *is preferred over other measures of generation time. It is the mean age of the mothers of a set of newborn individuals in a population with stable age distribution. The *T *value was 90 h for strain N2 and 84 h for strain MY6.

Life tables and fecundity schedules for the reproductive period of the two investigated *C. elegans *strains are given in Table 2. The total fertility rate (*TFR*, i.e., the total number of offspring a hermaphrodite would have, on average, if individuals were to live to the maximum age) of strain N2 was 295, and that of strain MY6 290. The net reproductive rate (*R*_0_, which depends on age-specific mortality rates) of N2 was 289, and that of MY6 291. This minute difference between the *TFR *and *R*_0 _values of the two strains is the result of the negligible juvenile and young adult mortalities (Table 2). Calculation of the intrinsic rate of natural increase (*r*_m_) yielded a value of 1.375 for strain N2 and 1.460 for strain MY6, corresponding to a population doubling time (*t *= ln(2)/*r*_m_) of 12.1 and 11.4 h, respectively.

## Discussion

### Method

Our study not only supplements previous investigations but also, more importantly, provides a comfortable approach to establish large cohort life tables at any desired temporal resolution. The hanging drop method overcomes several inconveniences associated with other methods employed in nematode life-cycle studies.

#### Solid Media

In the majority of life-cycle studies, *C. elegans *is maintained on NGM agar plates spotted with *E. coli *OP50. This is the standard method for cultivating *C. elegans*, but it holds several disadvantages. First of all, it is very difficult to control bacterial density on solid media. In the light of the obvious dependence of LHTs on food density, this appears rather unsatisfactory. One approach to overcome this limitation was developed by Tain et al. [9], who spread OP50 cells at different concentrations on NGM plates and killed them by exposure to UV light after varying time intervals. However, this approach only allowed a rough estimate of food conditions ("excess, high, and low"). Additionally, the effect of a diet consisting of dead bacteria is unclear. At least, *C. elegans *showed considerably reduced pharyngeal pumping rates when fed heat-killed OP50 [39]. The method used in the present investigation provides a fast and accurate method to adjust the medium to any desired food concentration. Moreover, when the influence of drugs, toxicants, or other compounds is to be tested, the test substance can be added to the medium immediately at the beginning of the experiment, thus minimizing its incorporation and metabolization by the bacteria.

A further difficulty in life-cycle experiments on solid media is the poor visibility of eggs and small juveniles on the uneven bacterial lawn, especially along the edge of a plate. Accordingly, Peters et al. [40] were confronted with a large error when counting eggs, which led them to conclude that egg counts on plates are likely to be substantial underestimates. The poor visibility of small juveniles within the bacterial lawn on agar plates is the probable reason that in most studies on the fecundity of *C. elegans *offspring was counted at the late juvenile to adult stage (e.g., [16,17,41]). However, early juvenile mortality and juveniles entering the dauer stage remain very difficult to record.

The problem can be reduced to some extent by the substitution of agar with gellan gum in the preparation of solid media [42]. The high transparency of such media considerably facilitates visual screening of culture plates. Moreover, as the strength of NGG media is determined by the concentration of divalent cations, low concentrations of the nontoxic chelating agent ethylenediaminetetraacetic acid (EDTA) break the bonds responsible for the gel matrix, yielding a liquid suspension that easily passes through sieves with a mesh size down to 10 *μ*m. This method permits the liquefaction of whole culture plates such that the nematodes can be readily extracted in virtually infinite numbers from cultures without adhering residues of culture medium [24]. However, in life-cycle experiments, the hanging drop method still has the advantage that all reproductive output is concentrated in one tiny drop of food medium that can be scanned quickly and accurately at high magnification.

In life- cycle studies carried out on agar plates, a substantial proportion of the population is usually lost or killed unintentionally during transfer or dies by desiccation after the worms crawl up the wall of the plate. This "lost" component may account for up to 58% of total replicates ([43]: note 29). But high artificial mortalities bias estimates of average lifespan, since long-living individuals have a higher probability of being censored from the survivorship data. In the present investigation, death by desiccation never occurred because the worms cannot overcome the hanging drop's surface tension. In total, only 2.1% (*n *= 3) of the total individuals were accidentally lost during handling, which is very low compared to the more than 3000 individual transfers conducted.

#### Liquid Media

Some of the inconveniences that accompany life-cycle experiments on solid media can be avoided by using liquid media, an approach that was established by Johnson & Wood [6]. Although the authors found that the lifespan of different stocks covaried, which they ascribed to "uncontrolled environmental effects", their approach established a basis for numerous seminal studies. Yet, the problem of environmental effects causing significant variation in fecundity and/or lifespan between replicates persisted (e.g., [5,44]). Friedman & Johnson [45] were forced to include a reference strain in all experiments due to this variation. Keightley et al. [17] additionally observed significant measurer effects, which they ascribed to differences in the relative levels of experience among the measurers carrying out the worm assays. It is known that the fecundity of *C. elegans *in liquid culture is generally lower than on solid media [5], a problem that Brooks [44] related to the lower oxygen availability in liquid. Other observations have included a far more rapid undulation behaviour ([46], after [47]) and reduced food consumption [48] in liquid media. Shook & Johnson [47] even found no correlation at all between survival on solid media and previous measures of survival in liquid media. In our laboratory, we have noted that it is difficult to adjust bacterial density in liquid culture because both bacteria and nematodes accumulate at the bottom of the vial, resulting in a higher *de facto *cell concentration in the nematodes' surroundings. In our opinion, the extreme conditions present within the slurry bacterial sediment might explain many of the difficulties associated with liquid culture.

As a matter of course, the high reproducibility of data obtained with the hanging drop method remains to be demonstrated. Yet, the fact that many of the above-listed problems associated with life-cycle experiments on solid or liquid media can be avoided in semi-fluid NGG make it a promising approach.

#### Temporal Resolution

Concerning the required temporal resolution of life-cycle studies, our data demonstrate the necessity of sufficient small age classes, at least during the first days of reproduction. In fast-reproducing species like *C. elegans*, minor differences in maturation time and other LHTs may be overlooked if the temporal resolution is set to one day or even longer. This can be illustrated with a simple example, taking as the basis the presented life table of strain N2 (Table 2), with the only modification being a slightly coarser temporal resolution at the very beginning of the reproductive period. Therefore, we pooled the fecundities of four 6-h age-classes (pivotal ages 56.5–74.5 h) into a single 24-h-age-class (pivotal age 65.5 h; net reproductive rate 14.2) and kept everything else equal. A calculation of the intrinsic growth rate based on this only slightly coarser life table yielded a value of *r*_m _= 1.406, corresponding to a 0.26-h underestimate of strain N2's population doubling time. This difference may initially be negligible but the difference between predicted and observed population sizes reaches 13% after only 4 days of unlimited growth. There are several scenarios thinkable in which a coarse temporal resolution in a life-cycle experiment will result in even more substantial errors of *r*_m_. Systematic errors that lead to over- or underestimation of the age of the cohort, such as may result from inaccurate estimates of hatching times, are even more problematic: When we introduced a systematic 12 h error into the life table of strain N2, *r*_m _rose to 1.640, resulting in a 189% difference between predicted and observed population sizes after 4 days of unlimited growth.

### Differences between strains N2 and MY6

An interesting result of this study is that strains N2 and MY6, isolated 700 km and 60 years apart from each other, do not differ with respect to most life-cycle parameters. No significant differences between the strains were found in key parameters such as total fecundity, lifespan, as well as age at and magnitude of maximum fecundity (Table 1). Likewise, the general shape of the two survivorship curves (Figure 1) was very similar, the minute differences being statistically non-significant. This finding is consistent with results from Johnson & Hutchinson ([5]: Table 1), who compared the lifespan of seven different *C. elegans *wild-type strains in liquid culture and concluded that all seven strains had similar life expectancies. Similarly, Sutphin & Kaeberlein [12] found no evidence that adaptation to the laboratory has altered the survival of *C. elegans *strain N2 compared to five different *C. elegans *wild-type strains grown on NGM agar. In the present study, the only significant differences between strains N2 and MY6 were a somewhat later deposition of the first egg (*T*_min_) and a somewhat faster hatching time (*T*_hatch_: Table 1) in N2. A longer hatching time, however, does not necessarily imply differences in the time span between fertilization and hatching. Instead, it may simply be due to the fact that strain N2 retains its eggs longer, depositing them at a later ontogenetic stage. In contrast, differences in *T*_min _are biologically important since time was measured between identical stages (freshly deposited eggs, see Methods) of successive generations. The observed 5.7-h-later time at which N2 first reproduced accounts for the calculated difference in the strains' population doubling times, 12.1 h for N2 and 11.4 h for MY6. This difference is quite small, especially when compared to the population doubling times of other free-living nematodes (e.g., 101 h in *Poikilolaimus*: see [24]). However, we can calculate the ratio of population sizes after some time *t*, given that both N2 and MY6 start with the same population size ([49]: p. 37):



It is clear that, as time progresses, the above ratio will increase, with MY6 becoming numerically more and more dominant in a theoretically combined population. After one week of unlimited growth, the ratio will be 1.8, rising to 3.3 after 2 weeks and 12.8 after one month. However, the assumption of extended periods of unlimited growth is unrealistic for natural populations: Within one month, an exponentially growing *C. elegans *MY6 population in stable age distribution (*r*_m _= 1.460) is theoretically capable of increasing (*N*_(*t*) _= *N*_(*0*)_*e*^*rt*^) by a factor of 10^19^. Converted into wet weight production, this means that, after one month, the progeny of a single *C. elegans *individual (wet weight roughly 1 μg) could potentially have a wet weight of 10^7 ^metric tons.

Why are the differences between the two strains negligible? As the Bristol variety of *C. elegans *had been in continuous laboratory culture for decades before being used to found the reference wild-type strain N2 [26], we expected distinct differences between this laboratory strain and a recently isolated natural strain such as MY6.

A possible explanation for the finding that strains N2 and MY6 nevertheless barely differed in their life histories can be found in the worm's particular androdioecious breeding system. Wild-type populations reproduce almost exclusively as self-fertilizing hermaphrodites, especially since the mating efficiency of the rare males is poor compared to a congeneric dioecious species [7]. In contrast to populations of outbreeding organisms, which maintain variant loci by selection for heterozygotes, selfing *C. elegans *populations are driven to homozygosity and consequently face strong selection against less-fit variants. This selection purges the population of deleterious recessive alleles [50] and consequently, wild-type *C. elegans *populations are already homozygous at most or all loci. Under these circumstances, it is plausible that selection for laboratory conditions and/or bottleneck effects act weaker in *C. elegans *than one would expect in genetically more diverse species. Indeed, laboratory populations of *C. elegans *face no or negligible inbreeding depression and crosses between laboratory strains show no heterosis (hybrid vigor) effects [4,5,7]. The homozygosity of the mainly self-fertilizing hermaphrodite *C. elegans *therefore well explains our finding that 60 years of laboratory culture apparently have not left a distinct trace in the Bristol strain's life cycle.

In conflict with our results, Chen et al. [25] found significant differences in several important demographic properties between strain N2 and a wild-caught *C. elegans *isolated from snails. Both total fertility and early survival of the wild-caught worm were lower; consequently, it obtained a considerably lower intrinsic growth rate than strain N2 (*r*_m _= ln(λ) = 1.249 vs. 1.348). The authors concluded that their results support the hypothesis that N2 has adapted to laboratory conditions. Apparently, there is a need for further studies, involving other strains from different geographic regions, to satisfactorily answer the question whether the Bristol strain N2 has undergone laboratory evolution.

In this context one should also mention that Johnson & Hutchinson [5] presented experimental evidence that the Berg BO (var. Bergerac) strain of *C. elegans *reproduces better in liquid culture than on solid agar plates. The authors argued that this characteristic might be the result of laboratory evolution, with an increased fecundity arising from the conditions under which this special stock was maintained i.e., axenic liquid culture. This interesting observation may offer opportunities to investigate the evolution of LHTs in the controlled environment of the laboratory.

### Comparison to earlier life-cycle studies

The results presented in this investigation are in good agreement with previous studies on *C. elegans*: The values for LHTs such as age at sexual maturity, mean and maximum lifespan, and total reproduction lie within the range reported by previous studies, as is discussed in detail below. However, a comprehensive review of the enormous number of studies dealing with various aspects of the life cycle of *C. elegans *is far beyond the scope of this paper. The vast majority of researchers in *C. elegans *are geneticists, and some 200 or more genes have now been found to cause hypomorphic (reduced function) mutations that extend life in the worm. According to Henderson et al. [22], *C. elegans *ageing studies have been summarized in more than 50 reviews alone, omitting other aspects of the *C. elegans *life cycle. We restrict the discussion of our results to a few general conclusions, keeping the focus on wild-type LHTs.

#### Survivorship

The survivorship curves of strains N2 and MY6 (Figure 1) did not differ significantly from each other and are strikingly similar to those presented by Johnson et al. ([30]: Figure 1A). On average, the two *C. elegans *strains in this study lived for somewhat longer than 2 weeks (Table 1), a lifespan that lies within the range reported by earlier studies. However, published lifespan estimates ([4-6,9,16,17,23,44,45,47,51-53], see Table 3) have suggested that *C. elegans *strain N2 tends to live longer in liquid (about 18.9 d) than on solid media (about 13.7 d). Our estimate (16.7 d) lies in the middle. Yet, there seems to be no simple relationship between culture medium and lifespan, since recombinant-inbred (RI) strains of *C. elegans *even showed a highly significant longer survival on agar than in liquid [47,53]. Generally, a long life is not necessarily an indicator of favourable conditions. For example, dietary restriction reduces fecundity and growth, but increases longevity in many organisms including *C. elegans *[9]. The reason for the relatively large variation in lifespan estimates between different studies is unclear, but may at least partly be related to suboptimal culture conditions and methodical problems, as discussed above.

**Table 3:** Exemplary studies reporting on lifespan and/or fecundity of *C. elegans*

	Strain	Medium	Lifespan [d]	Fecundity
Keightley et al. [17]	N2	solid	13.4	248
Halligan et al. [16]	N2	MYOB	11.9	258
Keightley & aballero [51]	N2	MYOB	14.0	255^**a**^
Chen et al. [52]	N2	NGM	14.8	285.6
Tain et al. [9]^**b**^	N2	NGM	15	244
Shook & Johnson [47]	N2	NGM	13.3	287
Johnson & Hutchinson [5]	N2	NGM	-	255
Hodgkin & Barnes [23]	N2	NGM	-	327
this investigation	N2	NGG	16.7^**c**^	291
Shook et al. [53]^**d**^	N2	NGM/S	15.7	254
Friedman & Johnson [45]^**d**^	N2	NGM/S	20.5 – 24.1	104 – 342
Johnson & Hutchinson [5]	N2	S	17.9	115
Brooks [44]	N2	S	15.2 – 20.6	94 – 238
Johnson & Wood [6]^**e**^	N2	S	18.2 – 22.7	-
Johnson & Hutchinson [5]	6 WI	S	14.8 – 20.8	-
Hodgkin & Barnes [23]	15 WI	NGM	-	235 – 353
Dolgin et al. [4]^**f**^	10 WI	NGM	13.3 – 14.9	228 – 262
this investigation	MY6	NGG	14.7^**c**^	289

All listed studies were conducted at 20°C with *E. coli* OP50 as food source. Underlined values indicate lifespans determined in liquid media.

**MYOB **= Modified Youngren's only bactopeptone (solid); **NGM **= nematode growth medium (solid); **S **= S basal medium plus cholesterol (liquid); **WI **= wild isolates, other than N2; ^**a **^value interpolated from Figure 2; ^**b **^values interpolated from Figs. 1A, 1C; ^**c **^includes time from egg deposition to hatching, see Table 1; ^**d **^fecundity was assayed on NGM, survival in S medium; ^**e **^*E. coli *B/r or OP50 used as food; ^**f **^values interpolated from Figs. 2A, 2C.

Although average and maximum lifespan estimates of *C. elegans *are naturally of tremendous importance in ageing research, they are relatively tedious from the ecologist's point of view because of the negligible juvenile and early adulthood mortality of *C. elegans*. All 141 juveniles observed in this investigation reached sexual maturity and 99% of them became older than 1 week (173.5 h, Table 2), i.e., until reproduction was essentially completed (Figure 2). But the intrinsic rate of natural increase (*r*_m_) is independent of lifespan as long as death occurs after the reproductive period. Life history theory suggests that the absence of significant selective pressure allows the accumulation of mutations that increase the probability of death beyond the reproductive period. In other words, selection favours those alleles that allow the individual to survive just long enough to reproduce competitively (disposable soma theory of ageing: see [54]). The evolution of genes influencing ageing is strongly dependent on, and in fact may be largely an accidental by-product of, selection of other LHTs [47].

#### Total Fecundity and Egg-Laying Rate

As Birch [3] pointed out, the intrinsic rate of population increase is determined to a much greater extent by the rate of oviposition at the very beginning of adult life than by the total number of eggs laid in the lifespan of the adult. With each successive day, the contribution of eggs laid to the value of *r*_m _decreases. In order to maximize population growth, a shortening of maturation time can thus be more advantageous than an increase in total egg production. Protandric *C. elegans *hermaphrodites face a trade-off between maturation time and total egg-production. Sperm is produced exclusively at the very beginning of the reproductive period and is subsequently used to self-fertilize oocytes. If the hermaphrodites do not mate, they face sperm limitation because many more oocytes than sperm are produced [55]. But an increase in sperm production is necessarily associated with a delay in the onset of egg production, which offsets the benefit of higher total fecundity. This trade-off was investigated by Hodgkin & Barnes [23], who provided empirical evidence that a mutant producing 50% more sperm is outcompeted by wild-type worms. The authors additionally investigated brood sizes of 15 recent natural isolates of *C. elegans *obtained from a variety of geographical locations. The strains were that similar in fecundity (Table 3) that the authors concluded that "a brood size of about 300 self-progeny is a universal optimum" for *C. elegans *populations all over the world. As a matter of fact, total fecundities of the two strains investigated in this study were very close to this number (N2 = 291, MY6 = 289).

Shook & Johnson ([47]: Table 2) found that *C. elegans *strain N2 needed 67.1 h from hatch to first reproduction, a value that differs by only 1.4 h from our results (Table 1: *T*_min _- *T*_hatch _= 65.7 h). However, strain MY6 only needed 57.4 h from hatch to first reproduction, an advantage that was only partly counterbalanced by its longer hatching time. As the two strains have almost identical total fecundities, the earlier onset of reproduction in MY6 cannot be the result of reduced sperm production. Interestingly, the egg-laying rate of strain MY6 showed a bipartite maximum, interrupted by a slightly decreased egg-laying rate (Figure 2). Considering the high number of replicates (*n *= 72), this temporary decrease cannot be regarded as an artefact and one might speculate that an adaptation of resource allocation in MY6 is somehow related to its faster maturation.

The maximum egg-laying rates observed in this investigation were somewhat above 140 eggs d^-1 ^(Table 1). Similar rates were reported by Tain et al. ([9]: Figure 1B) and Hodgkin & Barnes ([23]: p. 22). However, Johnstone et al. ([56]: Table 1a) reported considerably higher rates of 194 eggs d^-1^. The reason for this substantial difference may well be a behavioural peculiarity of *C. elegans *which we observed during our experiments: When a gravid worm is touched in order to remove it from the culture medium, it instantly empties its uterus and lays up to a dozen eggs. This 'stress deposition' potentially causes substantial overestimates of egg-laying rates, especially if only a single short time interval is investigated (3 h in [56]). We found that the use of an eyelash mounted on the tip of an applicator stick is very useful to reduce this source of error. With some practice, the worm is usually caught at the first attempt; eggs are thereby laid within the food medium adhering to the eyelash and transferred together with the worm.

#### Population Growth

Despite the wealth of excellent studies of survival, and the smaller number of studies of reproduction, there are almost no estimates of the population growth rate of *C. elegans *([25]: p. 1060). However, as shown in the seminal paper of Hodgkin & Barnes ([23], see above), the intrinsic population growth rate is a far more useful statistic than lifespan or fecundity alone, since *r*_m _integrates the entire age schedules of survival and fertility into a single measure. Intrinsic growth rates are also indispensable for reliable estimates of nematode biomass production, such as are needed in food-web analyses [57].

Vassilieva & Lynch ([15]: Table 1) calculated an intrinsic population growth rate of 1.35 for *C. elegans *(strain N2 on NGM, 20°C, OP50). However, this estimate may have been biased as the authors reported total fecundities of 170, which is less than 60% of the ~300 offspring usually produced by *C. elegans *([23], this investigation). A subsequent publication [58] had the same limitation (*r*_m _≈ 1.28; fecundity ≈ 175, interpolated from control line regressions in Figure 2). It was therefore surprising that the estimates of *r*_m _nonetheless differed only slightly from our calculations (N2: 1.375).

Keightley & Eyre-Walker ([59]: Figure 2a) also determined intrinsic growth rates of *C. elegans *strain N2. Unfortunately, their data are difficult to compare to our estimate because *r*_m _values were presented in terms of a frequency distribution of individual strains. However, their *r*_m _values were consistently higher than our estimate and in many of the lines (~40%) the intrinsic growth rates clustered around 1.53. In subsequent publications, Keightley and co-workers (e.g., [16,17,40]) also calculated intrinsic growth rates but did not present them because *r*_m _values were further transformed into the upper-order parameter "relative fitness." Although this fitness measure has certain advantages, it makes comparison between different studies impossible.

By using a Leslie matrix, Shook & Johnson ([47]: Table 2) estimated the expected population growth of *C. elegans *strain N2. From their derived factor "population growth" = e^100r ^= 686, the daily population growth rate can be calculated as ln(686)/100 × 24 = 1.567. This value is somewhat higher than our *r*_m _(1.375) and corresponds to a 1.5-h-shorter population doubling time. However, it was not clear whether the authors accounted for hatching times–apparently fecundity was determined as the number of fertile eggs laid within a certain time interval since hatching of the mother.

The most extensive study of *C. elegans*' LHTs conducted thus far was by Chen et al. [52], who monitored survival and reproduction of 1000 individuals of strain N2, maintained at 20°C on NGM seeded with OP50. The intrinsic growth rate (*r*_m _= ln(λ) = 1.348) reported by the authors was quite close to our estimate (1.375).

#### Conclusion

We found no evidence that adaptation to the laboratory altered the life history traits of *C. elegans *strain N2. The hanging drop method, in which nematodes are cultured in a drop of semi-fluid NGG, appears to be a useful strategy to assess the LHTs of the investigated strains. This approach has considerable potential in studies of other nematode species and in benthic meiofauna in general. Unpublished experiments in our laboratory indicate a high reproducibility; moreover, the method is simple enough to be applied by unpractised researchers. Given that further studies can validate the high reproducibility of data obtained with the hanging drop method, it may considerably simplify work in many *C. elegans*-related research fields.

## Authors' contributions

DM and FS contributed equally to the conception, design and accomplishment of the study. DM conducted statistical analysis and drafted the manuscript. WT conceived of the study, and participated in its design and coordination and revised the manuscript. All authors read and approved the final manuscript.
